# Pain management procedures used by dental and maxillofacial surgeons: an investigation with special regard to odontalgia

**DOI:** 10.1186/1746-160X-1-14

**Published:** 2005-12-22

**Authors:** Stefan Wirz, Hans Christian Wartenberg, Joachim Nadstawek

**Affiliations:** 1 Outpatients Pain Clinic – Department of Anaesthesiology and Intensive Care Medicine, University of Bonn, Sigmund-Freud-Str. 25, D-53105 Bonn, Germany

## Abstract

**Background:**

Little is known about the procedures used by German dental and maxillofacial surgeons treating patients suffering from chronic orofacial pain (COP). This study aimed to evaluate the ambulatory management of COP.

**Methods:**

Using a standardized questionnaire we collected data of dental and maxillofacial surgeons treating patients with COP. Therapists described variables as patients' demographics, chronic pain disorders and their aetiologies, own diagnostic and treatment principles during a period of 3 months.

**Results:**

Although only 13.5% of the 520 addressed therapists returned completely evaluable questionnaires, 985 patients with COP could be identified. An orofacial pain syndrome named atypical odontalgia (17.0 %) was frequent. Although those patients revealed signs of chronification, pain therapists were rarely involved (12.5%). For assessing pain the use of Analogue Scales (7%) or interventional diagnostics (4.6%) was uncommon. Despite the fact that surgical procedures are cofactors of COP therapists preferred further surgery (41.9%) and neglected the prescription of analgesics (15.7%). However, most therapists self-evaluated the efficacy of their pain management as good (69.7 %).

**Conclusion:**

Often ambulatory dental and maxillofacial surgeons do not follow guidelines for COP management despite a high prevalence of severe orofacial pain syndromes.

## Background

Many heterogeneous diseases lead to orofacial pain syndromes. Descriptions such as jaw (temporomandibular) joint pain, facial pain, and dental pain characterise these syndromes with regional anatomic descriptions. The International Headache Society [1-3] distinguishes orofacial pain syndromes from other painful conditions.

A perseverance of pain longer than 6 months and emerging signs of chronification, as a strong association with psychosocial problems, frequent changes of therapists, localisation of pain in other parts of the body, defines *chronic orofacial pain *[4-7]. Chronic orofacial pain very often has an economic impact on health care systems [8].

The female gender is affected, mostly. References show that prevalences of orofacial pain syndromes vary from a 6 month period prevalence of 12 to 22% to a 12 month prevalence of 20% [9-15].

The management of orofacial pain remains difficult. Often therapists cause a vicious circle by applying inadequate invasive treatment principles resulting in persistent pain conditions which then, for their part, force therapists to carry out further invasive procedures [8,16].

Only few investigations are publicized on the management of patients with orofacial pain treated by general physicians, pain therapists dental, or maxillofacial surgeons. A detailed assessment of *pain therapy *methods was not the objective of these studies [4,5,12,17]. Patients suffering from chronic orofacial pain are frequent in the Outpatients Pain Clinic of the University of Bonn, Germany. Previously, many of them had visited dental and maxillofacial surgeons. Contradictory, we knew only little about the diagnostic and therapeutic principles of these colleagues.

Therefore, this investigation aims to evaluate the ambulatory management of chronic orofacial pain syndromes by dental and maxillofacial surgeons within a defined German regional area, adjacent to our clinic. By assessing various diagnostic and therapeutic procedures we intended an evaluation of the realisation of the principles '*interdisciplinarity*' and '*multimodality*'. Furthermore, this investigation should describe demographic patterns of patients suffering from chronic orofacial pain syndromes.

## Methods

This investigation was designed as a descriptive, observational and cross-sectional case study.

In the 3rd quarter of the year 2001, the investigators sent questionnaires to all 508 dental surgeons and all 12 maxillofacial surgeons working in ambulatory capacities in a German county in the Rhine area containing 882,000 residents, called Rhein-Sieg-Kreis and Bundesstadt Bonn.

Questions referred to the number of patients suffering from orofacial pain during the 2nd quarter of the year 2001, their gender, age (expressed in decades), general medical characteristics, the classification of headaches and orofacial pain according the International Headache Society (IHS), and the aetiologies of pain. For the assessment of diagnoses, etiological factors, and the durations of pain we used standardised forms based on the IHS and the International Classification of Diseases, Version 10 (ICD-10).

Further points were the course of diagnostic procedures, including specialised diagnostic procedures, as radiological, neurophysiological, – especially electromyography -, and interventional procedures, as diagnostic blocks, or local anaesthesia. The investigators asked explicitly whether therapists knew or used visual analogue or numerical rating scales for assessing pain.

Other questions involved the use and prescription of analgesics – such as nsaids, opioids, anticonvulsants, antidepressants, and muscle relaxants – and surgical procedures, such as tooth extractions, or interventional procedures, such as local anaesthesia or sympathetic blocks. All therapists were given the opportunity to rate the efficacy of their management of orofacial pain syndromes in a three step scale ('poor', 'indifferent', 'good').

For the evaluation of the principle 'interdisciplinarity', all disciplines of therapists, including all medical and non-medical therapists, involved in the treatment of the patients experiencing orofacial pain were recorded.

Data were analysed descriptively by means of absolute numbers and percentages. Based on the total number of patients with chronic orofacial pain a three month prevalence was calculated for this sample.

## Results

Seventy-two of the 520 surveyed ambulatories returned completely evaluable questionnaires (13.5%). They reported 985 patients with orofacial pain being treated in the quarter in question. The calculated 3 month prevalence of orofacial pain based on 882,000 residents in the investigated county was 0.1%.

66.8% of patients with orofacial pain were female. Table 1 describes the distribution of the age decades of the sample. We found 81.1% of patients with an age between 20 to 60 years (mean age 31.9, minimum 7, maximum 88 years). No data on age was documented for 35 patients.

Patient diagnoses are listed in Table 2. Temporomandibular disorders (TMD), orofacial pain associated with headache syndromes and atypical odontalgia were very frequent.

Etiological factors of orofacial pain could be revealed in only 546 cases (55.4%). Detailed information is given in Table 3.

392 patients (39.8%) demonstrated co-morbidity. This involved orthopaedic (130 patients/33.2%), internal (127 patients/32.4%), psychosomatic (89 patients/22.7%), neurological (11 patients/2.8%), and psychiatric (14 patients/3.6%) causes.

Individual durations of orofacial pain were documented in 681 patients (69.1%). Pain persevered longer than 6 months in 61.1%, longer than 3 years in 21.3%, and longer than 5 years in 5.3%.

In most cases, diagnostics were carried out in the form of patient history and a general examination. Only 17.0% of the therapists knew of numerical rating or visual analogue scales as methods for assessing pain intensity. Only 7% used this device regularly. Specialised procedures, e.g. diagnostic local diagnostic blocks (4.7%), or neurophysiological procedures (5.7%), were rarely applied. However, radiological diagnostics were more frequent (52.4%).

Nevertheless, in 28.7% of patients the delay of diagnostic procedures lasted longer than one year. The diagnostics of three patients had not been completed over 15 years. Table 4 gives more details.

538 patients (54.6%) taking part in the survey had changed therapists before attending the ambulatory. 60.6% of these had changed their therapist more than three times.

Table 5 describes the enrolment of other disciplines in pain therapy. Further therapists were involved in diagnostics and therapy 761 times. Most frequently maxillofacial surgeons and pain therapists were involved, but the number of non-medical therapists exceeded the number of pain therapists. These consisted of physiotherapists and non-medical practitioners, a profession with permission to treat patients which exists in Germany only. Non-medical practitioners regularly use alternative medicine, such as traditional Chinese or Ayurvedic medicine, or homeopathy.

Although 985 patients suffered from chronic orofacial pain, only 635 received a documented treatment, as can be seen in Table 6. The use of further surgical procedures, e.g. tooth extraction, treatment of dental roots, was more frequent than the use of physiotherapy, analgesics or the treatment of a malocclusion. Eighty patients (8.1%) received nsaids, 9 (0.9%) opioids, 6 (0.7%) muscle relaxants and 4 (0.4%) anticonvulsants. The use of local anaesthesia was documented in 16 patients. Further interventional procedures, such as sympathetic blocks, were not used at all.

Responding colleagues self-evaluated the therapeutic efficacy of their methods applied in 885 cases (89.8%). In their perception, pain conditions of most patients improved. Pain rarely worsened, while no change was observed in nearly one-in-five patients, as listed in Table 7.

## Discussion

The use of a questionnaire is an established method for assessing the management of pain syndromes in patients, but the quota of respondents taking such an approach typically is small [5,6,12,17], as demonstrated in our investigation. Possibly, our questionnaire particularly induced responses from therapists with a very difficult clientele.

However, with nearly 1,000 patients currently being treated in 72 ambulatory dental and maxillofacial surgeries experiencing orofacial pain syndromes, the number was unexpectedly high compared to other studies [12]. On the other hand, the prevalence of orofacial pain extrapolated to all residents in the area of investigation was rather small, compared to other surveys which directly assess the population [4,9,12,13,15,18,19]. Contradictory to other references [11] and the general demographic data of this region, this investigation yielded a low rate of elderly people suffering from chronic orofacial pain, especially patients older than 60 years of age. However, the higher prevalence of orofacial pain in females, as described by other authors, was documented again [9,10].

We could not define etiological factors in nearly 50 % of patients with chronic orofacial pain. Surgical procedures, – especially if explorative and not correctly indicated -, revealed their harmful impact on chronification of pain.

The frequency of TMD diagnosis corresponded to other investigations, but the number of patients with the diagnosis *atypical odontalgia *including *phantom tooth *pain was unexpectedly high [14,15,18-22]. Deficits in knowledge of that pain condition, or effect of a selection (as mentioned above), might explain this. Some authors [8,14,21] could not identify atypical odontalgias because of methodological reasons, or just described it as dental pain, demonstrating an elevated prevalence [9,11]. Possibly, the epidemiological impact of atypical odontalgia is underestimated.

Atypical odontalgia is a severe and chronic pain disorder characterised by persistent pain with apparent clinical normal teeth. Clinical and radiographic examination does not reveal any pathologic findings. Neuropathic signs as allodynia and hyperalgesia are common and suggest a neuropathic origin of this pain. Heat, cold, pressure do not necessarily modulate pain. Local anaesthetics often have no impact. There is an elevated risk of chronification, as therapists often attempt vain interventional or surgical procedures [23-25].

Atypical odontalgia can be associated with atypical facial pain. Some authors consider atypical odontalgia to be a subgroup of atypical facial pain [20]. On the other hand, phantom tooth pain can be regarded as a special form of atypical odontalgia [26], a condition which occurred very often in this survey [27-29]. Furthermore, this investigation underlines the close relationship between both pain disorders.

The high number of patients involved in the working process underlines the possible socio-economic impact of orofacial pain. Unfortunately, socio-economic parameters (or at least of their being 'off work' due to orofacial pain) could not be documented. Nevertheless, patients with chronic orofacial pain revealed signs of chronification or an association with psychosomatic complaints. Other authors [15,18,28,29] also reported this association with headache syndromes.

This investigation showed that ambulatory therapists rarely followed the guidelines for the management of orofacial pain published by the American Academy of Orofacial Pain (AAOP) [30] and the German chapter of the International Association for the Study of Pain (Deutsche Gesellschaft zum Studium des Schmerzes – DGSS) [31]. Therapist skipped important and easily applicable devices as analogue scales for the assessment of pain intensity or diagnostic local anaesthesia. Despite a high co-morbidity with psychosomatic disorders, psychological or psychiatric diagnostics were omitted, causing further delays of the exact diagnostic process [7,17,23].

According to other references this investigation demonstrated another central neglect: the exclusion of an analgesic mediation in favour of surgery is an important etiological factor of the chronification of pain [31]. Although therapists have recognized its significance they to not perform the multimodal approach comprising different elaborated therapeutic strategies [6-8,16,17,21,23]. Treatment seldom comprises multidisciplinary aspects [17]: only few dentists and maxillofacial surgeons consulted colleagues from other disciplines, as pain therapists, neurologists, or psychiatrists.

Contradictory, dental and maxillofacial surgeons high-rated the efficacy of their procedures. The high number of 'successful' treatments contrasts with other references [5-7,23]. Possibly, causes are perceptive, communicative deficits, or administrative limitations of therapists treating severe and chronic orofacial pain syndromes.

## Conclusion

In the current management of patients suffering from orofacial pain syndromes ambulatory dental and maxillofacial surgeons ignore the principles of a multimodal and interdisciplinary pain therapy, despite their publication in various guidelines. A standardised concept of surgical, interventional and analgesic procedures has not been implemented so far. Therapists apply surgical procedures as tooth extractions or other surgical techniques rather than analgesics, minimal-invasive pain therapy, physiotherapy or other conservative procedures, although severe pain syndromes, such as atypical odontalgias, seem to be frequent in the sample population. Further prospective investigations and educational and communicative efforts should contribute to improving this situation.

## Competing interests

The author(s) declare that they have no competing interests.

## Authors' contributions

All authors were equally involved in the study design, data extraction, data analysis, and preparation of the manuscript.
